# Effect of home-based counselling on newborn care practices in southern Tanzania one year after implementation: a cluster-randomised controlled trial

**DOI:** 10.1186/1471-2431-14-187

**Published:** 2014-07-22

**Authors:** Suzanne Penfold, Fatuma Manzi, Elibariki Mkumbo, Silas Temu, Jennie Jaribu, Donat D Shamba, Hassan Mshinda, Simon Cousens, Tanya Marchant, Marcel Tanner, David Schellenberg, Joanna Armstrong Schellenberg

**Affiliations:** 1 London School of Hygiene and Tropical Medicine, Keppel Street, London, UK; 2 Ifakara Health Institute, Dar es Salaam, Tanzania; 3 Tanzania Commission for Science and Technology, Dar es Salaam, Tanzania; 4 Swiss Tropical and Public Health Institute, Basel, Switzerland; 5 University of Basel, Basel, Switzerland

**Keywords:** Newborn, Delivery of health care, Community health workers, Tanzania, Evaluation studies

## Abstract

**Background:**

In Sub-Saharan Africa over one million newborns die annually. We developed a sustainable and scalable home-based counselling intervention for delivery by community volunteers in rural southern Tanzania to improve newborn care practices and survival. Here we report the effect on newborn care practices one year after full implementation.

**Methods:**

All 132 wards in the 6-district study area were randomised to intervention or comparison groups. Starting in 2010, in intervention areas trained volunteers made home visits during pregnancy and after childbirth to promote recommended newborn care practices including hygiene, breastfeeding and identification and extra care for low birth weight babies. In 2011, in a representative sample of 5,240 households, we asked women who had given birth in the previous year both about counselling visits and their childbirth and newborn care practices.

**Results:**

Four of 14 newborn care practices were more commonly reported in intervention than comparison areas: delaying the baby’s first bath by at least six hours (81% versus 68%, OR 2.0 (95% CI 1.2-3.4)), exclusive breastfeeding in the three days after birth (83% versus 71%, OR 1.9 (95% CI 1.3-2.9)), putting nothing on the cord (87% versus 70%, OR 2.8 (95% CI 1.7-4.6)), and, for home births, tying the cord with a clean thread (69% versus 39%, OR 3.4 (95% CI 1.5-7.5)). For other behaviours there was little evidence of differences in reported practices between intervention and comparison areas including childbirth in a health facility or with a skilled attendant, thermal care practices, breastfeeding within an hour of birth and, for home births, the birth attendant having clean hands, cutting the cord with a clean blade and birth preparedness activities.

**Conclusions:**

A home-based counselling strategy using volunteers and designed for scale-up can improve newborn care behaviours in rural communities of southern Tanzania. Further research is needed to evaluate if, and at what cost, these gains will lead to improved newborn survival.

**Trial registration:**

Trial Registration Number NCT01022788 (http://www.clinicaltrials.gov, 2009)

## Background

Neonatal mortality (death with the first 28 days of life) most commonly occurs in South East Asia and sub-Saharan Africa, in the first seven days of life, and at home [1]. Reductions in childhood mortality will stall unless neonatal survival improves [1]. Hence, the World Health Organization (WHO) and global partners in maternal and child health recommend several low-cost measures including preventive practices, such as clean delivery, thermal protection and early and exclusive breast feeding, and interventions to manage complications, such as resuscitation and management of infections [2,3]. Reductions in newborn deaths of 41 to 72% have been predicted from universal coverage of such measures [4]. There is renewed interest in community health workers’ potential to increase coverage of these measures in communities served by primary health facilities [5,6].

Evidence from proof-of-principle trials in Southeast Asia indicates that home-based counselling in pregnancy and shortly after childbirth, combined with community-based treatment or referral of sick babies, can result in higher coverage of recommended newborn care practices [7], leading to reductions in neonatal mortality of between 34 and 62% [7-9]. Receiving a visit early in the postnatal period has been found to be associated with improved neonatal outcomes [10]. Two trials of community-based maternal and neonatal interventions in Southeast Asia implemented in programme settings have also reported increases in the practice of recommended behaviours [11,12], with one reporting a 15% reduction in neonatal mortality [12]. In Africa, a region suffering one million newborn deaths annually [13], only three similar interventions have been evaluated to date using an experimental design; all in programme settings. The Newhints trial in Ghana assessed the effect of community volunteers visiting women at home during pregnancy and the week after childbirth to promote essential newborn-care practices, weigh and assess babies for danger signs, and refer as necessary [14]. There were increases in the practice of recommended newborn care behaviours, including care-seeking for newborns (77% of sick babies in Newhints zones were taken to a health facility versus 55% in comparison zones) and initiation of breastfeeding within one hour of birth (49% versus 41%), but limited impact on neonatal mortality (risk ratio (RR) 0.9 (95% confidence interval (95% CI) 0.8-1.1). In Malawi, the MaiMwana study evaluated the effects of home-based counselling as well as another intervention - women’s groups – on maternal, neonatal and child health outcomes, including neonatal mortality rates, using a cluster randomised factorial design [15]. In the whole trial, although areas receiving home-based counselling reported higher levels of exclusive breastfeeding for the first six months (20%) compared to areas without counselling (9%; odds ratio (OR) 2·42 (95% CI 1·48–3·96)), there was no evidence of a reduction in neonatal mortality. In South Africa the evaluation of the Goodstart programme in Durban reported nearly a doubling of rates of exclusive breastfeeding (RR 1.92 (95% CI: 1.59–2.33)) at 12 weeks of age following a programme of pregnancy and post-natal home visits by community health workers [16].

Improving newborn survival is a current health priority for Tanzania to achieve the fourth millennium development goal [17]. In 2005 Tanzania had a neonatal mortality rate of 32/1000 live births [18], and the fourth highest number of neonatal deaths in sub-Saharan Africa [1]. The Improving Newborn Survival in Southern Tanzania (INSIST) project was conceived to develop, implement and evaluate a sustainable and scalable behaviour-change community intervention, with the aim of improving newborn survival in a region where neonatal mortality was higher than the national average [19]. Here we report the effect of the intervention on newborn care behaviours in the community one year after full implementation.

## Methods

The study is detailed in the protocol [20], and summarised below.

### Study design and area

The INSIST community intervention was implemented as a cluster-randomised trial in six districts of Southern Tanzania. Baseline data collected in five of those six districts in 2007 estimated the neonatal mortality rate at 34 per 1,000 live births (unpublished data). Intervention funding started in 2008. In 2009 the area comprised 132 wards, 720 villages and 3,428 sub-villages and had a population of around 1.2 million [21]. Each ward consists of an average of five villages, approximately 8,000 people, and 260 births per year. The area has a wide mix of ethnic groups. Common occupations include subsistence farming, fishing, and small-scale trading. Most rural roads are unpaved, some becoming impassable to motor vehicles in wet weather.

The public health system comprises a network of dispensaries, health centres and hospitals offering a varying quality of care [22]. The majority of health facilities are government-run; a health facility survey in the same districts in 2009 recorded four district hospitals, 15 health centres and 156 dispensaries [23]. Two regional hospitals just outside the study area provide referral care. Improving the quality of care in dispensaries and health centres for mothers and babies was originally intended to be part of the intervention, but resource limitations restricted this to implementation in just one study district. At the time the study started the majority of women attended antenatal care (88%) [22], around half (57%) delivered in a health facility [24] and no formal system existed for postnatal checks.

### Randomisation

In 2009 the research team allocated half of the 132 wards to receive the home-based counselling intervention in addition to routine care (n = 65), and half to continue to receive routine care only (n = 67). The 114 wards in the five districts with baseline data (Newala, Tandahimba, Lindi Rural, Ruangwa and Nachingwea) were randomised using implicit stratification to maximise balance in intervention and comparison groups. We listed the 114 wards in order of district, division, tertile of baseline neonatal mortality, and population, splitting them into 57 pairs. We allocated the wards in each ‘pair’ to intervention or control using random numbers generated by Microsoft Excel. This is equivalent to 57 tosses of a coin: the scheme has 2**57 = 10**17 realisations, so is highly unconstrained. For the district with no baseline data (Mtwara Rural) we listed the 18 wards by division, and then in alphabetical order within each division, and for each ‘pair’ of wards within this list we randomised the allocation of the wards in each ‘pair’ using random numbers generated by Microsoft Excel. This scheme has 2**9 = 512 realisations. There were no exclusion criteria for clusters, households, or women, after randomisation. All villages in intervention wards recruited volunteers to implement the counselling intervention. The nature of the intervention prevented blinding researchers, community members or health staff to the allocation.

### Design and implementation of the community intervention

The intervention, branded Mtunze Mtoto Mchanga (“protect your newborn baby” in Swahili), designed to be a sustainable and scalable part of the health system, was developed in 2008–9 on the basis of formative research [24,25]. Newborn care behaviours selected for targeting through the community intervention were in line with WHO recommended newborn care practices [2,3], and jointly agreed with key national stakeholders including regional health leaders, the Ministry of Health and Social Welfare, UNICEF, WHO and the Paediatric Association of Tanzania. Following development and piloting, in the first half of 2010 over 800 women who volunteered and were not currently involved in other community activities were recruited from and by their communities (two per village in intervention wards). They were trained for five days by their district health teams and followed-up in their villages after starting work as volunteers conducting home visits. All volunteers were working by June 2010. Volunteers were supported through quarterly review meetings with district health leaders, monthly contacts with village executive officers, who facilitated the link with the community, and with health facility staff, who provided technical support [26]. Two full-time staff from Ifakara Health Institute co-ordinated the community intervention through planning review meetings, compiling and reviewing monitoring data, and distributing behaviour change communication materials to districts.

For every pregnant woman identified in her village, a volunteer was expected to make three visits to her home during pregnancy and two in the early neonatal period, with additional visits for small babies (Table 1). The counselling focussed on one-on-one interaction between the volunteer and mother. Discussion with other family members involved in making decisions about childbirth and newborn care, including fathers and mothers-in-law, was encouraged. Behaviour change messages focused on hygiene during delivery, including gloves for those assisting childbirth, immediate and exclusive breastfeeding, and identification of and extra care for small babies. Additional behaviours promoted included: birth preparedness, with messages about the importance of health facility delivery and of preparing clean cloths, soap, gloves, a clean blade, a clean cord tie, and money; delayed bathing of the baby; and putting nothing on the cord. All counselling messages were introduced in pregnancy visits. Postnatal visits focused on reinforcing and supporting mothers to implement recommended practices directly applicable to the newborn. In addition, during postnatal visits to babies born at home, volunteers were trained to measure foot size as a proxy for birth weight, to counsel the mother to practise skin-to-skin care for babies who were smaller than usual and to refer very small babies to hospital in the early postnatal visits [27]. A picture-based card illustrating the counselling messages was used at each visit and left with each household to enable family members to aid retention of the information or, for those who were not present at the time of the volunteer visit, to receive the counselling messages. Volunteers used a locally-made doll (not left with the families) to demonstrate breastfeeding positioning and skin-to-skin care. Volunteers regularly reviewed antenatal care registers in order to identify new pregnant women in their village. Subsequent visits (except for the first postnatal) were scheduled at each counselling session. If a volunteer first visited a woman late in her pregnancy the gaps between the scheduled visits were reduced accordingly. If visits were missed, counselling messages at a subsequent visit were combined or adapted according to the schedule (Table 1) for the time of the visit in pregnancy or the neonatal period. Volunteers asked family members to notify them immediately after the birth in order to conduct postnatal visits. To support early postnatal home visits, facility staff gave mothers a delivery notification slip at discharge after delivery or when a baby was brought to a facility soon after birth, which staff advised families to take to the volunteer.

**Table 1:** Focus and timing of home visits for INSIST community intervention

**Visit**	**Timing**	**Key behaviours promoted**	**Additional behaviours promoted**	**Equipment**
1	As soon as pregnant woman identified	• Birth attendant should wash hands and wear gloves	• Birth preparedness: preparing for facility delivery and saving money; and preparing in case of unexpected home delivery, preparing clean cloths, soap, clean blade for cutting & clean thread for tying cord, gloves for birth attendant	Counselling card
2	Four weeks after visit 1	• Early and exclusive breastfeeding	• Check on birth preparedness issues from previous visit	Counselling card
3	At the beginning of 9^th^ month of gestation	• Early and exclusive breastfeeding including position	• Check on birth preparedness issues from previous visits	Counselling card with doll
		• In case of home birth:	• Warmth: immediate drying and wrapping, delayed bathing, keep the vernix	
		○ Birth attendant should wash hands and wear gloves, including while tying and cutting the cord		
		○ Identification of low birth weight babies using foot size as a proxy	• Danger signs for sick newborns	
		○ Immediate referral for very small or premature babies, and those who don’t cry	• In case of home birth, cord should be cut with clean blade and tied with clean thread	
		○ Skin to skin care for small babies		
4	Day of delivery	• Observe breastfeeding and counsel on positioning	• Check on warmth and knowledge of danger signs (as above)	Counselling card – measure foot size using scale
		• Reminder of exclusive breastfeeding	• Put nothing on cord	
		• In case of home birth:		
		○ Identification of low birth weight babies using foot size as a proxy		
		○ Immediate referral for very small or premature babies		
		○ Skin to skin care for small babies		
5	Third day after delivery	• Observe breastfeeding and counsel on positioning	• Put nothing on the cord	Counselling card
		• Reminder of exclusive breastfeeding		
1st extra visit for small baby	Day after visit 5	• Skin to skin until the baby doesn’t want to be carried skin to skin		Counselling card
2nd Extra visit for small baby	Day after visit 6	• Skin to skin until the baby doesn’t want to be carried skin to skin		Counselling card

### Sampling

The household survey sample size was based on the number of women age 13–49 who had given birth in the year preceding the survey, which the baseline survey suggested was approximately 10% [24]. We assumed one woman of reproductive age resided in each household and aimed to visit 40 households per ward (n = 131; one ward did not participate), which gave a sample size of 5,240 households and an estimated 524 reportable deliveries. The study was powered to detect a 15 percentage point change in the practice of early breastfeeding initiation, with 95% confidence and 80% power, including a design effect of 1.5 to account for clustering.

We used multi-stage sampling to select households. In stage one, for the districts with baseline data we selected one sub-village from within each ward with probability proportional to size (PPS). For Mtwara Rural district, with no baseline data, we obtained numbers of households in each village, which serves as a proxy for population. We ran the same PPS method, this time selecting villages. In stage two, within each village a sub-village was selected by simple random sampling, and 40 households were selected for interview using a modified EPI-type sampling approach that gave all households an equal chance of selection [28]. The method, used by the research team in previous surveys, is detailed elsewhere [22] and summarised here. In the centre of each sub-village the supervisor threw a pen to choose a random direction. (S)he walked in the direction indicated until the edge of the sub-village, sketching a map of and numbering all the households passed. One of these households was selected at random as the first household. At this household, the supervisor threw a pen to choose a random direction, and walked in that direction until (s)he came to another household, which was the second household, and so on until 40 households were counted. If there was a junction in the path, a pen was thrown again to select from the choices available. Villages were visited one day before the survey interviewers arrived, and an invitation letter left in each of the selected households.

### Data collection, processing and quality control

The household questionnaire was developed from tools used by the Demographic and Health Surveys (DHS) [18], Newhints [14] and the baseline household survey [24]. Questions asked to household heads determined his/her occupation, household members and assets. Female residents aged 13–49 years at the time of the survey were asked about their birth history. Those who had delivered a live baby in the year preceding the survey were asked about their pregnancy, delivery and newborn care practices, and receipt and content of any home-based counselling during pregnancy and the neonatal period. The questionnaire was pre-tested in one sub-village using printed forms. Pendragon Forms 4.0 software (http://pendragonsoftware.com/) was used to develop a modular questionnaire data entry template. For data collection, the questionnaire was loaded onto Dell Axim X51 personal digital assistants (PDA)s with 64 MB RAM. The PDA version of the questionnaire was piloted in one ward before the main survey started.

Data were collected in August and September 2011 by trained interviewers who visited selected households, sought written informed consent for survey participation, and recorded responses on PDAs. If household heads refused to participate no other household members were approached. If no household members were present at the time the interviewer visited, the household was visited again later the same day. Households were not replaced in cases of refusal or absence. Logical checks and skip patterns took place at data entry. Digital data records were locked after leaving each household. Data were downloaded to laptop computers and daily summary reports produced to evaluate completeness and consistency.

Field supervisors undertook a number of quality control activities. Firstly, each supervisor accompanied interviewers to three households each day. Secondly, they revisited households where interviewers had reported that there were no residents, or the household heads refused participation. Lastly, two households were revisited daily and a small number of interview questions repeated, the responses to which were compared with those collected by the interviewer.

### Data analysis

Data were analysed at the individual level using Stata v12. We calculated means and proportions of respondent characteristics, intervention coverage, delivery characteristics and newborn care behaviours. To estimate the size of the effect of the intervention, logistic regression analysis was used to calculate the ORs of women reporting behaviours in intervention wards compared with those from comparison wards, using svy commands to account for the clustered study design and multi-stage sampling.

Receipt of the intervention was defined as reporting being visited by a volunteer who had used one of the Mtunze counselling tools (card or doll), to exclude other community health activities. A wealth index score, as a measure of socio-economic status, was constructed for each household using the first principal component of ten household assets and characteristics [29], namely ownership of a radio, bicycle, telephone, poultry, livestock and the home, household connection to an electricity supply, roofing material, cooking fuel and source of income. Households were ranked according to this total wealth score and divided into quintiles.

To investigate the effect of the intervention on childbirth and newborn care practices we compared mothers’ self-reported behaviours (Table 2) for those who gave birth in the preceding year in intervention and comparison areas, using the allocation given at the sub-village level (intention-to-treat analysis). The primary outcomes were breast feeding within an hour of delivery, birth attendants for home deliveries washing hands before childbirth or wearing gloves, and babies fed only breast milk in the first three days. Secondary outcomes were the other behaviours promoted during counselling to maximise newborn health, e.g. skilled attendance for childbirth, birth preparedness (for home deliveries), immediate drying and wrapping of the baby, clean cord care and delayed bathing of the baby [3]. Although a key behaviour of the intervention, this study was not powered to detect changes in the levels of identification and provision of extra care for small babies. We assumed that childbirth in health facilities took place on a clean surface, that the birth attendant had clean hands or wore clean gloves, and that the cord was cut with a clean blade and tied with a clean thread, so these behaviours were only asked about and reported for home deliveries. The data analyst was masked to the cluster allocation until analysis was complete.

**Table 2:** Outcome measures

**Outcome category**	**Practice**	**Timing of practice**	**Measured for which babies?**
Primary	Baby breastfed within one hour of birth	Newborn care	All
	Birth attendant washed hands with soap before childbirth or wore gloves	Childbirth	Home birth
	Baby fed only breast milk in the first three days	Newborn care	All
Secondary	Childbirth in a health facility	Childbirth	All
	Childbirth with a skilled attendant	Childbirth	All
	Prepared soap	Childbirth	Home birth
	Prepared new or washed cloth for drying baby	Childbirth	Home birth
	Prepared cloth or mat for childbirth	Childbirth	Home birth
	Cleaned floor where childbirth to take place	Childbirth	Home birth
	Prepared new or washed cloth for wrapping	Childbirth	Home birth
	Had plan in case of emergency childbirth	Childbirth	Home birth
	Attendant had clean hands during childbirth	Childbirth	Home birth
	Baby had cord cut with new or sterilised blade	Newborn care	Home birth
	Baby had cord tied with new thread	Newborn care	Home birth
	Baby dried <5 minutes after birth	Newborn care	All
	Baby wrapped <5 minutes after birth	Newborn care	All
	Baby bathed at least six hours after birth	Newborn care	All
	Baby had nothing applied to umbilical cord	Newborn care	All

### Ethical approval and consent procedures

The study was part of INSIST (http://www.clinicaltrials.gov, NCT01022788), and was approved by the review boards of Ifakara Health Institute, the Medical Research Coordinating Committee of the National Institute for Medical Research, Tanzania Commission for Science and Technology, and the London School of Hygiene and Tropical Medicine, UK.

Prior written consent to approach village leaders was obtained from each district council. Village and sub-village leaders gave verbal consent for data collection to proceed before any households were approached. The head of each household gave written informed consent to participate. In the absence of the household head, another adult resident was approached to give consent. If no adult residents were present the household was revisited later in the day. If a household head refused to participate or adults were absent no replacement households were sought. The consenting adult resident was asked about the members of his/her household and the ownership of household assets. All females age 13–49 in consenting households gave their individual verbal informed consent before being interviewed.

## Results

### Respondents

**Figure 1:**
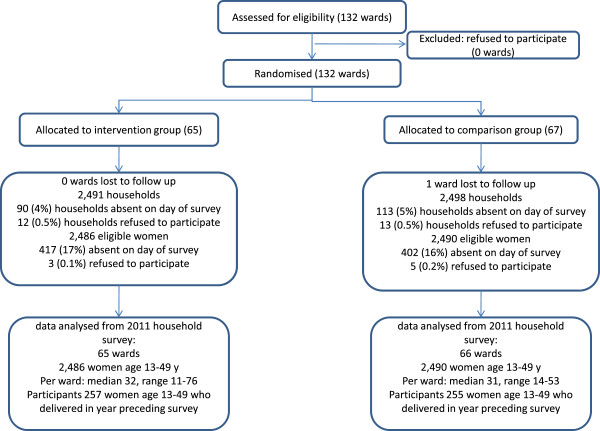
Trial profile.

We visited 131 of 132 wards, as one ward did not participate in the survey. In each of these wards we visited one sub-village. We visited a total of 5,217 households. Although 5,240 households were expected, in ten sub-villages data were obtained from fewer than 40 households. Some sub-villages were small: in six sub-villages we obtained data from 39 households, in two 38 households, and in one 37 households. In one sub-village data from ten households were lost. In the households visited, 4,989 (96%) household heads agreed to participate, 2,491 in intervention areas and 2,498 in comparison areas (Figure 1). These households comprised a population of 19,475 people, of whom 4,976 were women aged 13–49. Of these, 4,157 (84%) were available for interview, 4,149 (83%) agreed to participate and 3,199 (78%) had ever delivered a baby. There were 512 women (257 in intervention areas, 255 in comparison areas) from 128 of the sub-villages who had delivered 521 live babies (including nine pairs of twins) since 1st August 2010 and went on to answer detailed questions about their most recent birth, and who comprise the respondents in these analyses.

Background characteristics were similar in intervention and comparison areas. Respondents had a mean age of 28 years (standard deviation (sd) 7.2 years) and had completed a median of seven years of education (range 0–17 years, Table 3). Household heads were mainly from the Makonde ethnic group. There were 12 neonatal deaths in intervention areas and 9 in comparison areas (neonatal mortality rates of 47/1000 live births (95% CI 24–91) and 35/1000 live birth (95% 19–64) respectively, p = 0.521) in the year preceding the survey.

**Table 3:** Respondent characteristics

**Characteristic**	**Intervention (I) wards (N = 257)**		**Comparison (C) wards (N = 255)**		**Percentage points difference (I-C)**
	**n**	**%**	**n**	**%**	
Wealth quintile					
1 (Poorest)	52	20	49	19	1
2	52	20	49	19	1
3	53	21	50	19	2
4	46	18	58	23	-5
5 (Wealthiest)	52	20	51	20	0
Ethnic group of household head					
Makonde	140	54	141	55	-1
Mwera	82	32	72	28	4
Makuwa	8	3	13	5	-2
Yao	10	4	7	3	1
Other	17	7	22	9	-2
Years of Education					
0-6	96	37	103	40	-3
7	142	55	141	55	0
8-17	19	7	11	5	2
Age					
15-19	32	12	39	15	-3
20-24	70	27	83	33	-6
25-29	58	23	45	18	5
30-34	40	16	50	20	-4
35-39	38	15	26	10	5
> = 40	19	7	12	5	2

### Coverage of home-based counselling intervention

Seventy-three percent (187/257) of women in intervention areas reported receiving a counselling visit, and seven percent (18/255) in comparison areas (Table 4). Women most commonly received their first counselling visit at five months gestation (sd 1.6 months).

**Table 4:** Coverage and implementation of home-based counselling intervention

**Intervention event**	**Intervention wards**		**Comparison wards**		**Percentage points difference (I-C)**	**OR (95% CI)**	**p**
	**n (N)**	**%**	**N (N)**	**%**			
Woman ever received an Mtunze visit	187 (257)	73	18 (255)	7	66	35.2 (19.4-63.6)	<0.001
Woman received an Mtunze visit during pregnancy	187 (257)	73	18 (255)	7	66	35.2 (19.4-63.6)	<0.001
Woman received an Mtunze visit after childbirth	118 (257)	46	8 (255)	3	43	26.2 (11.6-59.3)	<0.001

Women reported receiving a mean of 2.4 counselling visits (standard error (SE) 0.1). Most commonly women reported receiving three visits in pregnancy (87/187, 47% of those reporting any visit, range 1–8) and one in the neonatal period (86/187, 46% of those reporting any visit, range 0–3). Of women reporting receiving visits, 14% (26/187) reported receiving the full complement of at least five visits. Eighteen percent (34/187) of women reported receiving the first postnatal visit within two days of childbirth; most commonly it was received three days after birth (inter-quartile range 2–5 days).

Eleven of the 16 women in intervention areas who reported that their baby was smaller than normal at birth received counselling visits; none reported receiving more than two postnatal visits. None of the 11 women in comparison areas who reported that their baby was smaller than normal at birth received counselling visits.

### Childbirth characteristics and newborn care behaviours

The majority of women gave birth in a facility (69%) or with a skilled attendant (71%). Similar proportions of women reported childbirth in a health facility or with a skilled attendant in intervention and comparison areas (both OR 1.4 (95% CI 0.9-2.3)) (Table 5).

**Table 5:** Childbirth characteristics, all deliveries

**Childbirth characteristic**	**Intervention wards**		**Comparison wards**		**Percentage points difference (I-C)**	**OR (95% CI)**	**p**
	**n (N)**	**%**	**n (N)**	**%**			
In a health facility	187 (256)	73	166 (254)	65	8	1.4 (0.9-2.3)	0.14
With a skilled attendant	189 (255)	74	171 (254)	67	7	1.4 (0.9-2.3)	0.16

The majority of women giving birth at home reported that each of the birth preparedness activities was undertaken, with little difference between intervention and comparison areas (Table 6). More women in intervention than comparison areas reported that the cord was tied with a clean thread (69% versus 39%, OR 3.4 (1.5-7.5), all used new thread). There was little evidence of differences between the groups with regard to whether or not the birth attendant had clean hands or the cord was cut with a clean blade.

**Table 6:** Childbirth practices, home deliveries

**Practice**	**Intervention wards**		**Comparison wards**		**Percentage points difference (I-C)**	**OR (95% CI)**	**p**
	**n (N)**	**%**	**n (N)**	**%**			
Prepared soap	63 (68)	93	80 (88)	91	2	1.3 (0.4-4.0)	0.70
Prepared new/washed cloth for drying baby	61 (68)	90	82 (88)	93	-3	0.6 (0.2-2.0)	0.43
Prepared cloth or mat for birth	51 (53)	96	69 (73)	95	1	1.5 (0.2-10.2)	0.69
Cleaned floor where birth to take place	50 (67)	75	66 (88)	75	0	1.0 (0.5-2.0)	0.96
Prepared new/washed cloth for wrapping baby	65 (69)	94	80 (88)	91	3	1.6 (0.5-5.3)	0.42
Had plan in case of emergency delivery	49 (68)	72	63(88)	72	0	1.0 (0.5-2.2)	0.95
Attendant washed hands before childbirth or wore gloves	55 (68)	81	65 (88)	74	7	1.5 (0.6-3.6)	0.37
Baby had cord cut with new or sterilised blade	65 (68)	96	81 (88)	92	4	1.9 (0.5-7.8)	0.39
Baby had cord tied with clean thread	46 (67)	69	34 (87)	39	30	3.4 (1.5-7.5)	0.003

A minority of women reported that their babies were dried (33%) or wrapped (20%) within five minutes of delivery, and reported rates were similar in intervention and comparison areas (Table 7). The majority of women reported delaying the baby’s first bath by at least six hours, and this was more commonly done in intervention (81%) than comparison (68%) areas (OR 2.0 (95% CI 1.2-3.4)). Although breastfeeding within an hour of birth was reported by less than a third of women, with little evidence of a difference between intervention and comparison areas, exclusive breastfeeding in the first three days after delivery was reported by the majority of women in all areas, and more commonly in intervention (83%) than comparison (71%) areas (OR 1.9 (95% CI 1.3-2.9)). Applying nothing to the cord was commonly reported, more so in intervention (87%) than comparison (70%) areas (OR 2.8 (95% CI 1.7-4.6)). One hundred and three respondents reported applying substances to the cord; most commonly oil (20%) and herbs (15%).

**Table 7:** Newborn care practices, all deliveries

**Practice**	**Intervention wards**		**Comparison wards**		**Percentage points difference (I-C)**	**OR (95% CI)**	**p**
	**n (N)**	**%**	**n (N)**	**%**			
Baby dried <5 minutes after birth	84 (253)	33	85 (253)	34	-1	1.0 (0.6-1.5)	0.89
Baby wrapped <5 minutes after birth	56 (255)	22	45 (254)	18	4	1.3 (0.8-2.1)	0.26
Baby bathed at least 6 hours after birth	207 (255)	81	173 (254)	68	13	2.0 (1.2-3.4)	0.007
Baby breastfed within 1 hour	68 (249)	27	53 (251)	21	6	1.4 (0.9-2.1)	0.11
Baby fed only breast milk in first 3 days	206 (249)	83	178 (250)	71	12	1.9 (1.3-2.9)	0.002
Nothing applied to umbilical cord	222 (255)	87	179 (255)	70	17	2.8 (1.7-4.6)	<0.001

No adverse events as a result of the intervention were reported.

## Discussion

This cluster-randomized controlled trial of a community-based home counselling intervention by volunteers found high levels of coverage for a programme designed for implementation at scale: around three-quarters of women in intervention areas received a counselling visit during pregnancy, and half in the early postnatal period. One year after full implementation, in intervention areas, four recommended newborn care practices were more common than in comparison areas: delaying the baby’s first bath by at least six hours, exclusive breastfeeding in the three days after birth, putting nothing on the cord, and, for home births, tying the cord with a clean thread.

Other evaluations of similar interventions implemented in the programme setting have reported wide variations in intervention coverage. Our coverage of pregnancy visits (76%) was comparable with the Newhints study in Ghana (72%) [14], higher than reported in Pakistan (63%) [12] but lower than in a study from Bangladesh (reported receipt of at least one antenatal visit was >90% at most recent measurement) [11]. Our coverage of postnatal visits (47%) was lower than reported from Ghana (63%) [14] and in Bangladesh (80%) [11], but higher than reported in Pakistan (24%) [12]. The MaiMwana study in Malawi reported 55% of women in intervention areas had received a counselling visit at any time when asked at one month post-delivery [15], compared to 78% in our study.

Despite variations in the newborn care practices included in the published trials of similar interventions, some comparisons can be made with our findings. Exclusive breastfeeding was reported by a majority of women in both the INSIST (during the three days after childbirth) and Newhints (at 26–32 days old) studies, with a slightly higher proportion reporting the practice in intervention than comparison areas in both cases (INSIST 83% versus 71%, OR 1.9 (95% CI 1.3-2.9), Newhints 86% versus 80%, RR 1 · 10, 95% CI 1 · 04–1 · 16) [14]. A trial in Uttar Pradesh found much lower rates of pre-lacteal feeding in areas implementing community-based promotion of essential newborn care compared to comparison areas (38% versus 80%, rate ratio 0.49 (95% CI 0.42-0.57)) [7]. The proportion of respondents reporting delaying the first bath until at least six hours after delivery was between 12 and 23 percentage points higher in intervention areas in the evaluations in Pakistan, Ghana and our study in Tanzania (Pakistan: 50% versus 27%, p = 0.008. Ghana: 41% versus 29% rate ratio 1 · 65, 95% CI 1 · 27–2 · 13. INSIST: 81% versus 68%, OR 2.0, 95% CI 1.2-3.4) [12,14].

While there is evidence of association between clean cord practices and improved neonatal survival [30,31] several studies evaluating the impact of combined packages of care did not report changes in cord care practices [14,15], or presented only data on the use of a clean instrument to cut the cord [7,9,11]; a behaviour already known to be practised by the vast majority of the population in our study area [24]. Thus there is little evidence from similar trials with which we can compare the increases in rates of applying nothing to the cord and use of clean cord ties found in this study.

National data show some similarities with and some differences from our study findings. The Tanzania DHS 2010 found that 68% of deliveries in the Southern Zone took place in facilities [32], which is comparable with the proportion found in this survey (69%) and reflects a large increase since this study’s baseline survey in 2007 (41%) [24]. Nationally around half of babies were reported to have been breastfed within an hour of birth [32]. This is considerably higher than in our study. The more specific recording of the timing of feeding initiation in our questionnaire compared to that used by the DHS may explain this difference, and has been discussed previously [24]. Finding comparable rates of exclusive breastfeeding in the first three days between DHS 2010 and this study, where similar wording was used, supports this argument.

The coverage of some behaviours, such as wrapping and drying of the baby within five minutes of birth and breastfeeding within an hour of birth, remained low in intervention areas. Formative research in the study area suggested that the attention of birth assistants remained with the mother until the placenta was delivered in home births, which may explain these findings [33]. Further encouragement of facility deliveries, or the presence of an additional birth attendant to assist the baby at home deliveries may help improve thermal care of newborns.

Although the proportion of women delivering at home who reported that their birth attendant had clean hands was higher in intervention than comparison areas, the difference was inconclusive. It may be that the intervention had no effect on hand cleaning practiced by birth attendants, or there was insufficient power to detect the difference in the practice of this behaviour that related only to home births.

Receipt of counselling visits may have been over-reported in intervention areas. Volunteer counsellors were residents of their village and pregnant women were identified to be approached to be visited for counselling through antenatal registers: women who chose not to receive counselling visits were still likely to have been aware of the programme in their community and may have reported receiving a counselling visit. By randomising at ward level, the study reduced intervention ‘leakage’ to comparison areas: although 18 (7%) women in comparison areas reported receiving an intervention counselling visit, only four (2%) had a study counselling card. These could have been women living close to intervention areas, or women who had moved to be with relatives for childbirth. Some of the reported counselling visits in both intervention and comparison areas could have been linked to other community activities.

This study showed improvements in newborn care behaviours with home-based counselling in programme settings, but the changes may have been greater with improved intervention implementation. For example, early postnatal visits are associated with improved neonatal outcomes [10], but in our study fewer than half of women in intervention areas received a postnatal visit and a fifth of women reporting receiving any counselling visit were visited within two days of childbirth. There are many possible reasons for this. Volunteers were trained by staff at the district level, so volunteers’ knowledge of the counselling programme may have varied. Motivational activities and a supervision programme were included in the design of the counselling programme, which are known to help to maintain volunteer work standards and coverage [34]. However, these may have not been sufficient or novel enough to sustain volunteers over long time periods. These were reviewed regularly during implementation and steps taken to improve them where needed. A substantial change to the supervision procedures in 2011 increased the frequency of supervision, and could improve counselling coverage and quality, although it was implemented too late for its effect to be measured by this evaluation [26]. In the context of increasing health facility deliveries, early postnatal visits may have been difficult to complete and measures to facilitate them ineffective.

For analysis we assumed that facility birth would involve adequate basic obstetric care, including clean delivery surfaces, the cord being cut and tied with clean instruments, and immediate wrapping and drying of the baby. However health system weaknesses mean this may not always be so. Evidence from a health facility survey in the study area found many instances of missing or recently out of stock items for delivery, including examination gloves and cord ligatures, particularly in dispensaries [23]. Such weaknesses and the limited quality improvement work undertaken alongside the community intervention means care of mothers and newborns in health facilities is likely to remain a barrier to improved newborn survival.

There is some evidence from sub-Saharan Africa that clean delivery practices, early and exclusive breastfeeding and early skin-to-skin care are associated with improved newborn survival [35]. While the target behaviours, and content and mode of delivery of the messages would need to be adapted to the local setting in order to implement a similar intervention beyond this region of Tanzania, this study contributes to the small but growing robust evidence base that home-based counselling implemented at scale in the community can improve newborn care practices in low-resource African settings with high levels of neonatal mortality [14,15]. A meta-analysis of trials of home-visit strategies in Africa and Asia found overall a 12% (95% CI 5–18) reduction in neonatal mortality [14]. Therefore future research needs to establish if the behaviour change reported here is sufficient to reduce neonatal mortality. Furthermore, the impact of the number and timing of counselling visits on mortality rates should be assessed. Lastly, the quality, acceptability and cost effectiveness of the counselling intervention need to be evaluated to understand the process of behaviour change and sustainability of the intervention.

## Conclusions

A home-based counselling strategy to promote recommended newborn care implemented by volunteers and designed for scale within the health system can improve newborn care in rural communities in southern Tanzania. Further research is needed to evaluate if, and at what cost, these gains will lead to improved newborn survival.

## Competing interests

The authors declare that they have no competing interests.

## Authors’ contributions

SP analysed the data, interpreted the study findings and co-led the writing of the manuscript. FM trained the data collectors and interpreted the study findings. EM trained the data collectors, monitored data collection for the whole trial and helped to clean the data. ST trained the data collectors, programmed the PDAs for data collection and cleaned the data. JJ and DDS trained the data collectors. HM, DS and MT conceived the INSIST study, wrote the study protocol and designed the evaluation questionnaire. JAS conceived the INSIST study, wrote the study protocol, designed the evaluation questionnaire, interpreted the study findings and co-led the writing of the manuscript. All authors read and approved the final manuscript.

## Pre-publication history

The pre-publication history for this paper can be accessed here:

http://www.biomedcentral.com/1471-2431/14/187/prepub
